# Learning curve analysis of transurethral thulium fiber laser enucleation of the prostate with preserving urethral Mucosa at the prostatic apex

**DOI:** 10.3389/fsurg.2025.1646928

**Published:** 2025-09-16

**Authors:** Dawei Xie, Yirui Wei, Weifeng He, Hao Wang, Pushen Yang, Liyang Wu, Jianwen Wang

**Affiliations:** Department of Urology, Beijing Chaoyang Hospital, Capital Medical University, Beijing, China

**Keywords:** ThuFLEP, BPH, learning curve, linear regression, feasibility

## Abstract

**Objective:**

To introduce a unique surgical technique, analyze perioperative data to demonstrate the safety of Thulium Fiber Laser Enucleation of the Prostate (ThuFLEP) with preservation of the urethral mucosa at the prostatic apex, and construct a learning curve to assess its feasibility and potential for broader clinical application is the aim of this study.

**Methods:**

From June 2020 to June 2024, a urologist at Beijing Chaoyang Hospital, with no prior ThuFLEP experience, was trained under the supervision of an experienced chief physician. A retrospective analysis of 100 Benign Prostatic Hyperplasia (BPH) patients treated with ThuFLEP was conducted. Perioperative data were analyzed, including prostate volume, operative time, and enucleation weight. Statistical methods included *T*-tests, chi-square tests, and linear regression. Learning curves were constructed using Loess regression, with box plots visualizing differences in operative time, efficiency, and enucleation ratio efficacy. Postoperative follow-up assessed changes in IPSS, Qmax, and urinary incontinence.

**Results:**

The learning curve analysis showed a significant reduction in operative time after 56 cases. Linear regression indicated a decrease in operative time (*R* = −0.5, *p* < 0.01) and improvements in efficiency (*R* = 0.14, *p* < 0.01) and enucleation ratio efficacy (*R* = 0.41, *p* < 0.01) with increased experience. Comparing the first and last 50 cases, operative time and enucleation efficiency significantly improved (*p* < 0.01). Postoperatively, 31% of patients experienced incontinence one week after catheter removal, but all recovered within 12 weeks. IPSS scores improved by 13.67 ± 3.99 points.

**Conclusions:**

ThuFLEP with urethral mucosa preservation improves efficiency, reduces operative time, and has a manageable learning curve. Attention to complications and structured mentorship are crucial for successful implementation.

## 1 Introduction

BPH is a common condition in elderly men, with its incidence increasing markedly with age. Data show that the prevalence reaches nearly 8% in men over 40 and exceeds 90% in those over 90 (1). Benign Prostatic Obstruction (BPO) is a common cause of bladder outlet obstruction in elderly men, leading to lower urinary tract symptoms (LUTS) such as frequent urination, urgency, and difficulty, which significantly impair quality of life. While both medical and surgical treatments are available, surgery has become the preferred option in recent years due to its rapid and substantial therapeutic effects (2, 3).

Common surgical techniques include Transurethral Resection of the Prostate (TURP), Holmium Laser Enucleation (HoLEP), Thulium Laser Enucleation (ThuLEP), Transurethral Incision (TUIP), and minimally invasive procedures like UroLift and Rezum (4–6). The thulium fiber laser is non-inferior to the holmium laser in terms of bleeding, catheterization time, and operative time (5, 7–9). Furthermore, preserving the urethral mucosa at the prostatic apex has been adopted by many centers to further minimize complications (10).

However, the learning curve for Transurethral Thulium Fiber Laser Enucleation of the Prostate (ThuFLEP), particularly with urethral mucosal preservation, has not been thoroughly explored (11). This study analyzes 100 ThuFLEP cases performed by a single surgeon at Beijing Chaoyang Hospital from June 2020 to June 2024, aiming to introduce a unique surgical technique, analyze perioperative data to demonstrate the safety of Thulium Fiber Laser Enucleation of the Prostate (ThuFLEP) with preservation of the urethral mucosa at the prostatic apex, and construct a learning curve to assess its feasibility and potential for broader clinical application.

## 2 Methods

### 2.1 Study design

Between June 2020 and June 2024, a urologist from Beijing Chao-Yang Hospital, Capital Medical University, with experience in transurethral plasma vaporization of the prostate but without prior experience in ThuFLEP, was trained in the procedure, specifically preserving the urethral mucosa at the prostatic apex, under the supervision of Chief Physician. A retrospective analysis was conducted on data from 100 patients with BPH treated by this surgeon.

Inclusion criteria required a preoperative diagnosis of BPH with absolute or relative indications for surgery. Exclusion criteria included prostate cancer, urethral stricture, neurogenic bladder, and bladder neck contracture. Perioperative data were collected as detailed in Table 1. Both total prostate volume and transition zone volume were measured via MRI. If the patient's total prostate-specific antigen (TPSA) exceeded 4.0 ng/ml, with an MRI PI-RADS score over 3, or if a palpable nodule was detected during a digital rectal examination, a biopsy was performed to confirm benign status before inclusion in the study.

**Table 1:** Perioperative basic information of patients.

Variable	Mean ± SD	Median (min ∼ max)
Age (years)	71.84 ± 7.78	72.50 (51.00 ∼ 96.00)
TPSA (ng/ml)	5.82 ± 5.17	4.21 (0.30 ∼ 25.59)
FPSA (ng/ml)	2.83 ± 7.18	1.23 (0.07 ∼ 58.77)
Total Prostate Volume (cm^3^)	77.51 ± 40.12	71.15 (20.00 ∼ 333.60)
Transition Zone Volume(cm^3^)	48.48 ± 30.24	41.85 (7.00 ∼ 210.00)
Transition Zone/Total Ratio	0.60 ± 0.14	0.61 (0.22 ∼ 0.89)
Pre-op Catheter (weeks)	3.23 ± 8.78	0.00 (0.00 ∼ 56.00)
Qmax (ml/s)	5.60 ± 3.29	5.30 (0.50 ∼ 23.40)
IPSS	23.26 ± 3.56	24.00 (14.00 ∼ 32.00)
Total Surgery Time (min)	91.90 ± 41.93	87.50 (30.00 ∼ 290.00)
Morcellation Time (min)	10.46 ± 12.93	8.00 (2.00 ∼ 120.00)
Operative Time (min)	81.44 ± 36.26	78.50 (20.00 ∼ 170.00)
Enucleation tissue Weight (g)	34.23 ± 27.00	29.40 (5.00 ∼ 170.30)
Enucleated tissue weight/Transition Zone Volume	0.66 ± 0.22	0.63 (0.23 ∼ 1.57)
Enucleated tissue weight/surgical time	0.43 ± 0.30	0.33 (0.07 ∼ 1.43)
Enucleation Ratio Efficacy	0.01 ± 0.01	0.01 (0.00 ∼ 0.05)
Post-op IPSS	9.59 ± 2.78	10.00 (4.00 ∼ 16.00)
Follow-up Qmax (ml/s)	17.21 ± 5.38	18.90 (5.20 ∼ 25.90)
IPSS change	−13.67 ± 3.99	−14.0 (−24 ∼ −4)

### 2.2 Surgical procedure

**Figure 1:**
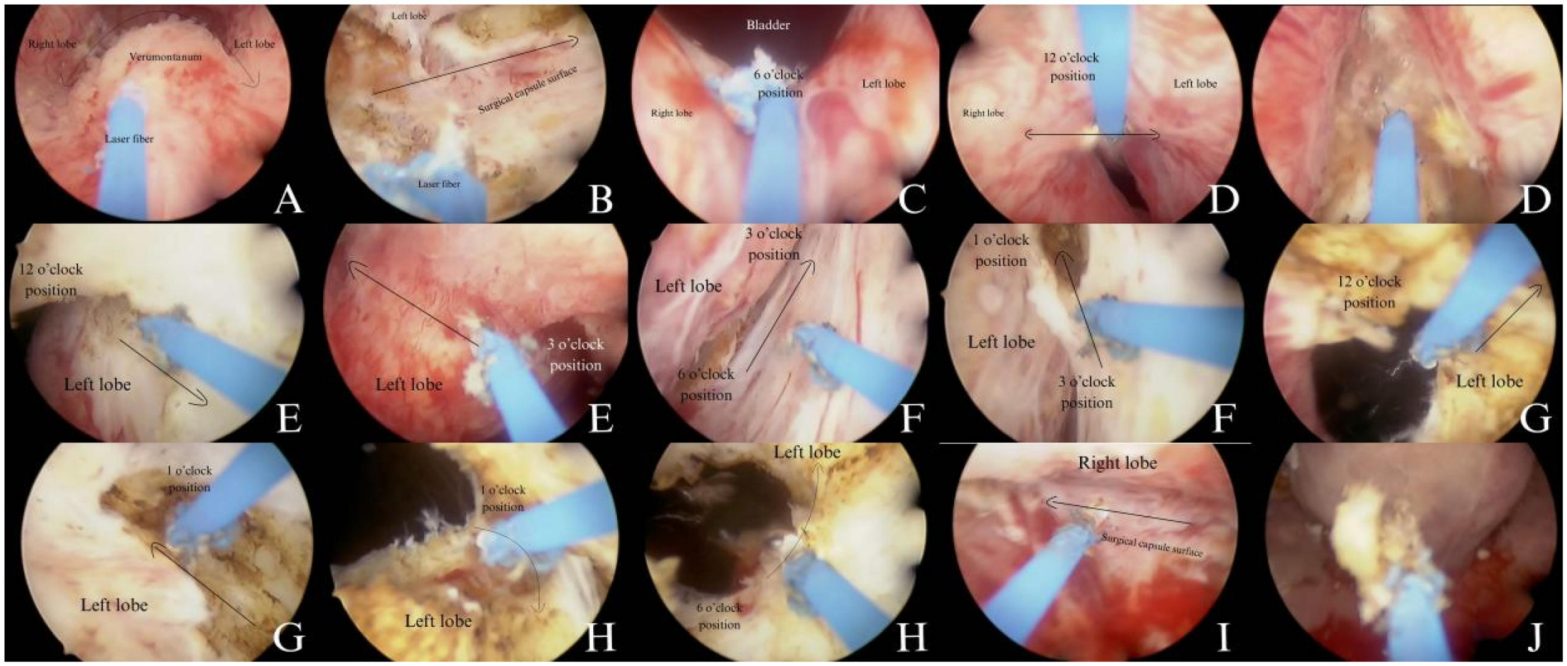
A detailed explanation of the surgical procedure.

The surgical procedure, ThuFLEP with mucosal preservation at the prostatic apex, was performed as follows:

A. The mucosa and glandular tissue were incised in an inverted U-shape near the seminal colliculus.
B. The laser was used to create a gap between the left lobe and the surgical capsule.
C. A groove was made from the 6 o'clock position of the bladder neck to the prostatic apex, separating the lobes.
D. A second groove was created from the bladder neck to the apex at the 12 o'clock position, widening towards the left lobe.
E. The mucosa between the 12 o'clock and 3 o'clock positions was interrupted to avoid sphincter damage while preserving the left apex mucosa.
F. The left lobe was enucleated along the 6-3-1 o'clock positions, with meticulous hemostasis throughout.
G. The transverse incision connected the left lobe at the 1 o'clock and 12 o'clock positions, freeing it.
H. The left lobe was pushed into the bladder from the 1 o'clock, 3 o'clock, and 6 o'clock positions.
I. The right lobe was treated using the same steps as the left lobe.
J. A morcellator was then used to fragment the excised tissue. The procedure preserved the mucosa from the 9 o'clock to 12 o'clock to 3 o'clock positions at the prostatic apex (Figure 1).

### 2.3 Calculation and graphing methods

Perioperative data for 100 patients were summarized in Table 1. The learning curve for surgery time was plotted using Loess regression, with data smoothed by the Savitzky-Golay filter, first derivative analysis, and segmented regression to define key stages. Linear Regression of Operative Time, Efficiency, and Enucleation Ratio Efficacy was performed, with scatter plots illustrating these relationships. An independent *T*-test and chi-square test compared perioperative data between the first and second groups of 50 patients. Boxplots were used to display differences in operative time, efficiency, and enucleation ratio efficacy. Postoperative follow-up tracked changes in International Prostate Symptom Score (IPSS) and maximum urinary flow rate (Qmax), as well as the incidence and recovery of urinary incontinence, graded via the Modified Sphincteric Incontinence Grading Scale (MSIGS). Follow-up was conducted weekly for up to 12 weeks. The number of patients with pre-op catheter is 37.

All statistical analyses were performed using R version 4.2.3 and *P*-values below 0.05 were considered statistically significant.

### 2.4 Ethical approval

This study was conducted in accordance with the Declaration of Helsinki and approved by the Ethics Committee of Beijing Chao-Yang Hospital, Capital Medical University. The thulium laser equipment used was provided by Raykeen Company (Chinese National Medical Device Registration Certificate No. 20193010884), with enucleation parameters set at 1.5J energy and 50W power, and hemostasis parameters at 1.0J energy and 20W power. The laser fiber had a diameter of 750 μm and a core diameter of 550*μ*m.

## 3 Results

**Figure 2:**
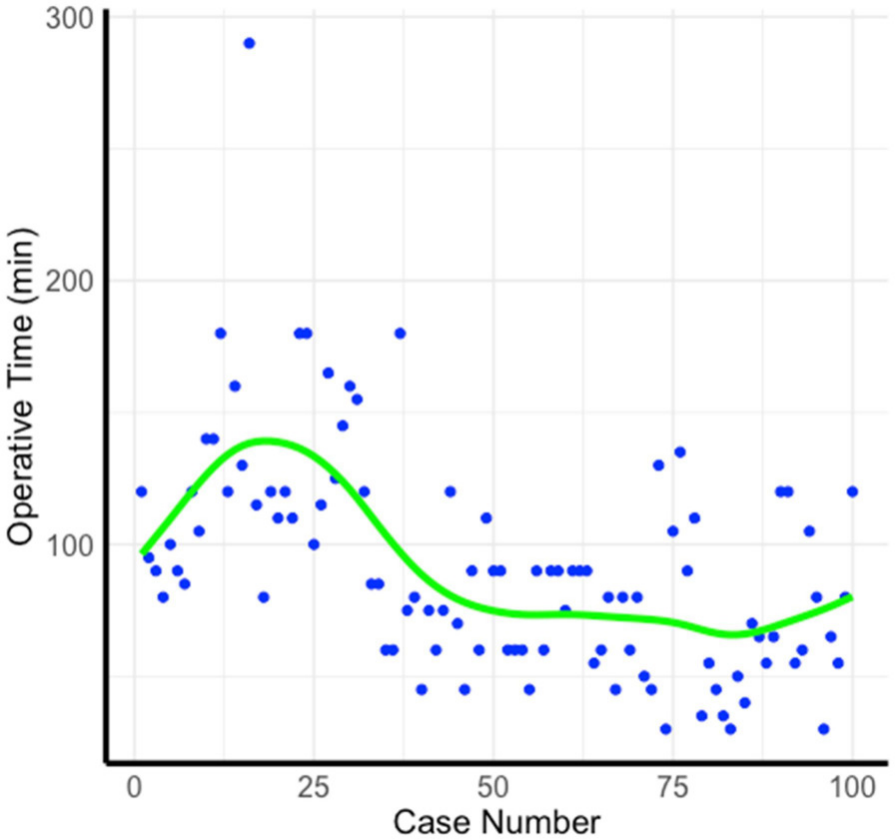
Learning curve. The figure illustrates the operative time across 100 cases. Each blue dot represents the operative time for an individual case, with the green Loess regression line showing the overall trend as the number of cases increases.

The analysis of the learning curve identified five distinct stages, each with varying time trends and operational complexity. In the Initial Stage (Cases 1–12), surgery time fluctuated significantly due to the surgeon's lack of proficiency, with longer operative times. The 12th case marked a turning point, after which surgery time decreased. In the Rapid Improvement Stage (Cases 12–19), surgery time dropped sharply as critical skills were acquired, stabilizing by the 19th case. The Gradual Stabilization Stage (Cases 19–30) showed a slower decrease as proficiency increased. During the Stable Stage (Cases 30–56), surgery time became consistent with reduced variability, reflecting the standardization of techniques. By the 56th case, surgery times reached a low, stable level, marking the Proficient Stage (Cases 56–100), with minimal fluctuations, indicating full proficiency (Figure 2).

**Figure 3:**
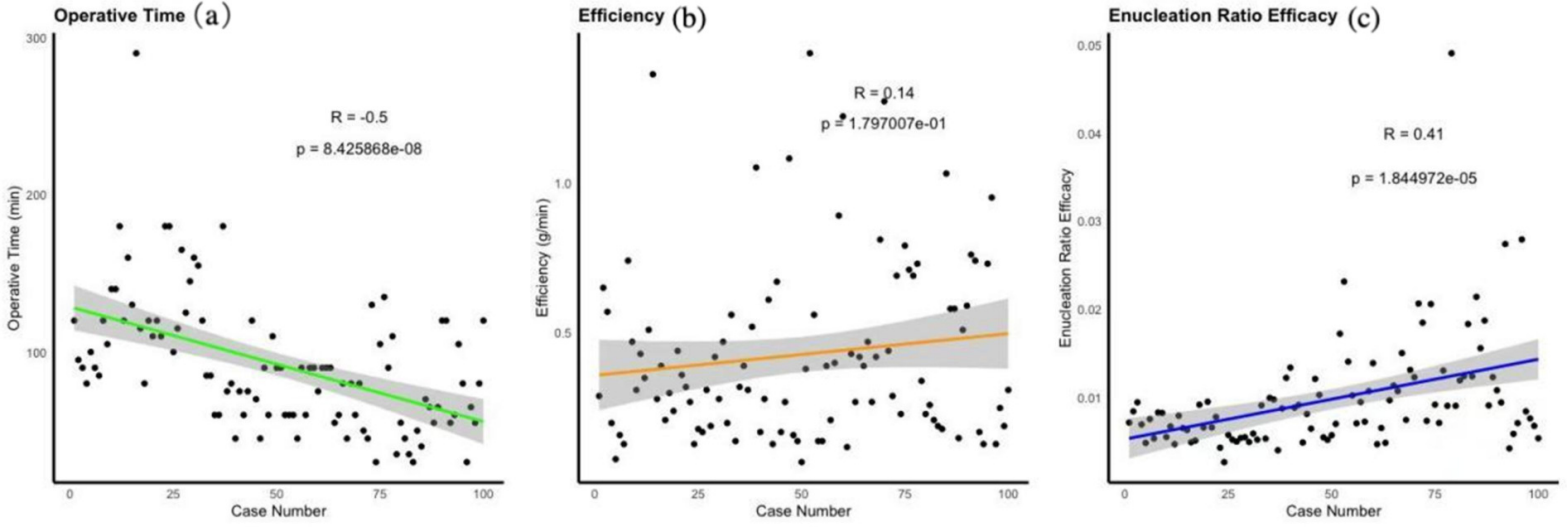
Representing changes over time using linear regression. **(a)** Linear Regression of Operative Time, **(b)** Linear Regression of Efficiency, **(c)** Linear Regression of nucleation Ratio Efficacy.

We calculated and plotted linear regression graphs for Operative Time, Efficiency, and Enucleation Ratio Efficacy against the number of cases. Scatter plots illustrated the relationships for each patient. The regression between Operative Time and case number showed a significant downward trend (*R* = −0.5, *p* < 0.001). Efficiency demonstrated a slight upward trend (*R* = 0.14, *p* < 0.001), and Enucleation Ratio Efficacy increased with the number of cases (*R* = 0.41, *p* < 0.001) (Figure 3).

**Figure 4:**
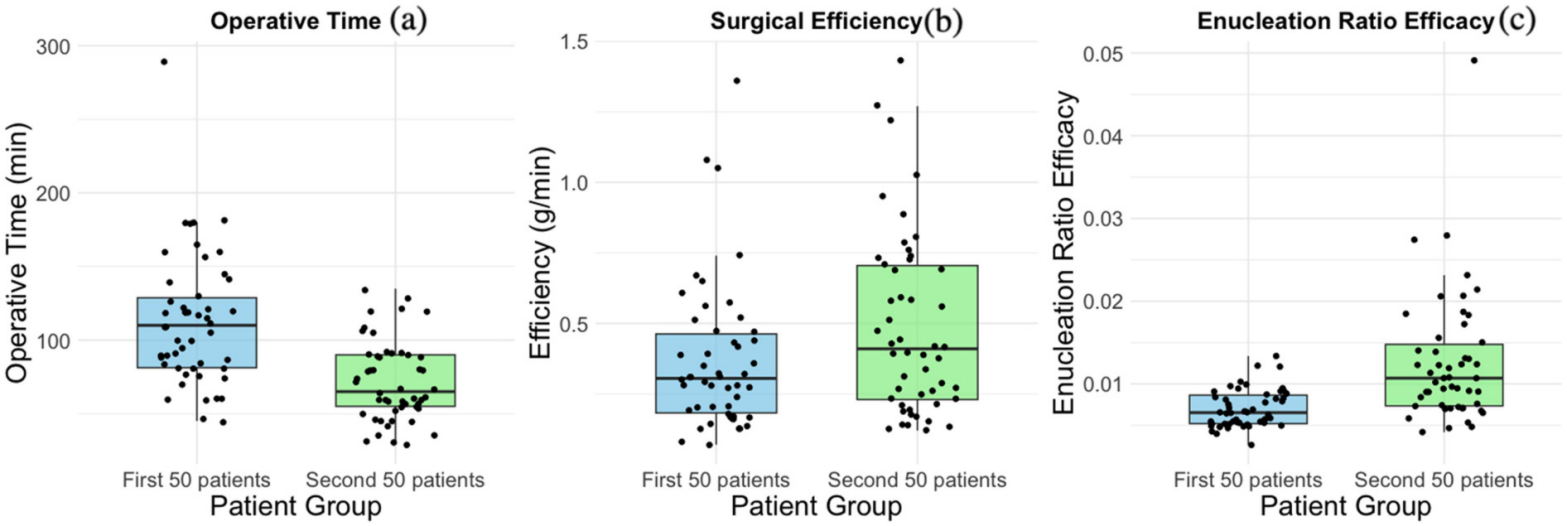
Comparison between the first 50 cases and the second 50 cases. **(a)** Boxplots of Operative Time, **(b)** Boxplots of Efficiency, **(c)** Boxplots of Enucleation Ratio Efficacy.

The learning curve turning point was identified at 56 cases, leading to a comparison between the first and subsequent 50 patients using an independent sample *T*-test and chi-square test (Table 2, Figure 4). Significant improvements were observed in operative time (*p* < 0.01), enucleation efficiency (*p* < 0.05), and enucleation ratio efficacy (*p* < 0.01), with faster operative times and increased efficiency in the latter group. Additionally, the prevalence of age and diabetes was higher in the second group (*p* < 0.05). Other perioperative factors, including prostate volume, morcellation time, and complications such as postoperative bleeding and re-catheterization, showed no significant differences between the two groups.

**Table 2:** Perioperative basic information of patients of the first 50 and second 50.

Parameters	First 50	Second 50	*p*-values^a^
Age (years)	70.00 ± 6.61	73.68 ± 8.47	<0.05
TPSA (ng/ml)	5.53 ± 4.03	6.07 ± 6.07	0.596
FPSA (ng/ml)	3.17 ± 8.68	2.53 ± 5.40	0.659
Total Prostate Volume (cm^3^)	83.21 ± 45.31	71.82 ± 33.65	0.157
Transition Zone Volume (cm^3^)	52.40 ± 32.10	44.57 ± 28.04	0.197
Operative Time (min)	112.20 ± 44.39	71.60 ± 27.23	<0.01
Morcellation Time（min）	11.51 ± 16.84	9.66 ± 7.43	0.479
Enucleation tissue Weight（g）	37.05 ± 28.74	32.91 ± 25.49	0.448
Enucleation Efficiency（g/min）	0.37 ± 0.26	0.53 ± 0.37	<0.05
Enucleation Ratio efficacy（g/ml/min）	0.007 ± 0.001	0.013 ± 0.001	<0.01
Hypertension (n)	27 (23)	27 (23)	1.000^b^
Diabetes (n)	6 (44)	16 (34)	<0.05^b^
Previous Prostate Biopsy (n)	5 (45)	10 (40)	0.262^b^
History of Prostate Medication(n)	31 (19)	29 (21)	0.838^b^
History of Indwelling Catheter(n)	19 (31)	19 (31)	1.000^b^
Bladder Left Wall Mucosal Injury(n)	0 (50)	1 (49)	1.000^b^
Postoperative Bleeding (n)	2 (48)	0 (50)	0.495^b^
Re-catheterization within 24 h (n)	1 (49)	1 (49)	1.000^b^

^a^ P-value obtained using independent sample *T*-test.

^b^ P-value obtained using chi-square test.

TPSA, Total Prostate-Specific Antigen; FPSA, Free Prostate-Specific Antigen; Enucleation Ratio Efficacy = (Enucleation Ratio)/(Enucleation Time); Enucleation Ratio = Enucleation tissue Weight/Transition Zone Volume.

**Figure 5:**
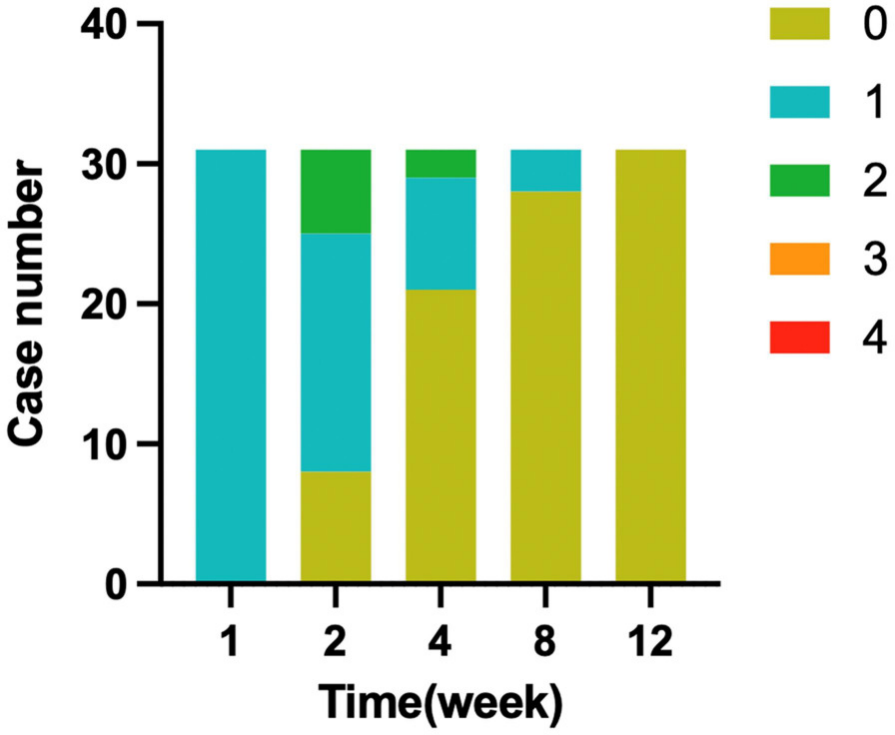
Urinary incontinence recovery after catheter removal.

Postoperative follow-up included monitoring changes in IPSS, Qmax, and urinary incontinence, graded using the MSIGS scale (0–4). Of 100 patients, 31 experienced leakage one week after catheter removal. Recovery rates improved over time, with 8 patients recovering by two weeks, 13 by four weeks, 7 by eight weeks, and 3 by twelve weeks, as shown in Figure 5. In 38 patients monitored for Qmax, 36 showed improvement, while 2 experienced a decrease in flow rate. The mean IPSS score decreased by 13.67 ± 3.99 compared to preoperative values, indicating significant symptomatic relief.

## 4 Discussion

Benign prostatic hyperplasia (BPH) is a common, progressive condition in elderly men that significantly impacts quality of life due to lower urinary tract symptoms (LUTS). While medication offers symptomatic relief, transurethral prostate enucleation has consistently demonstrated superior long-term outcomes (12). Our results align with previous findings, showing significant improvements in quality of life and clinical metrics such as the International Prostate Symptom Score (IPSS) and Bladder Outlet Obstruction Index (BOOI) (3, 13). Although holmium laser and bipolar plasma have been widely used (10, 14, 15), our findings suggest that the thulium fiber laser provides superior improvements in urinary flow and residual urine reduction (9). These advantages, particularly the precision and hemostatic control offered by thulium, underscore its potential as a preferred energy source in BPH surgery.

Transurethral thulium fiber laser enucleation demonstrates excellent vaporization and hemostasis capabilities (5). Its precision in cutting reduces the need for excessive mechanical manipulation, minimizing tissue trauma and lowering the risk of sphincter injury. The shallower working depth further enhances its safety profile. To reduce postoperative urinary incontinence, we preserved the apical mucosa during the procedure. By excising the prostate apical mucosa while maintaining the 12 o'clock mucosa and glandular tissue, we mitigated the risk of sphincter damage from mucosal traction. This approach shows promise in improving surgical outcomes and reducing complications, offering valuable insights for refining enucleation techniques and guiding future practice.

While learning a new surgical technique, the physician is also performing transurethral plasma resection of the prostate. Moreover, upon completing the learning of the ThuFLEP surgery, the physician is also able to perform transurethral plasma enucleation of the prostate. This is thanks to the familiarity with the prostate's anatomical structure gained while learning the enucleation surgery, which aids in surgical advancement.

The learning curve summarized in this article, based on 100 cases, began to decrease around 25 cases, plateaued around the 35 cases and stabilized after 56 cases. By comparing the first 50 cases with the second 50 cases, we believe that inexperienced surgeons, under the guidance of a single mentor, can reach a plateau in operative time after performing 50–60 surgeries. This is similar to the results of other studies (16, 17). It should be noted that in this study, none of the patients were switched to TURP midway; all began and ended with the Thuflep surgical method. This, in contrast to other studies, also illustrates the ease of learning and safety of Thuflep (18).

Our analysis demonstrated a significant reduction in operative time as surgical experience increased (*R* = −0.5, *p* < 0.01), with a corresponding improvement in efficiency (*R* = 0.14, *p* = 0.18) (Figure 3). Furthermore, the enucleation ratio efficacy showed a continuous increase (*R* = 0.41, *p* < 0.01), contrary to prior reports suggesting stabilization after around 30 cases (19). This sustained improvement may be attributed to the structured mentorship provided during the learning process, allowing for ongoing refinement of surgical techniques. These findings underscore the critical role of mentorship in enhancing operative performance, supporting conclusions from previous studies regarding the importance of guided learning in improving surgical outcomes (20).

We assessed whether preoperative transrectal prostate biopsy, a history of prostate medication, or indwelling catheter use influenced operative time, enucleation efficiency, and postoperative complications. No significant correlations were found, consistent with prior studies (21). One patient experienced a bladder mucosal injury at a prominent trabecula, with a morcellator-related injury risk typically ranging from 1.2% to 5.23% (22). This occurred during the surgeon's stable proficiency phase, suggesting it may not be linked to technical skill but rather anatomical factors. Studies suggest ultrasound guidance during morcellation could mitigate this risk, warranting further research (23). Two patients in the early learning phase (first 50 cases) experienced postoperative bleeding, while no such complications occurred in later cases, reflecting improved proficiency. Additionally, two patients required re-catheterization within 24 h postoperatively, both of whom had preoperative urinary obstruction and high residual urine volumes. Their urinary function recovered within a week, indicating that re-catheterization was likely related to pre-existing conditions rather than surgical technique.

Our comparison of preoperative and postoperative IPSS and Qmax changes aligns with previous studies, showing significant improvements following thulium fiber laser prostate enucleation (9). Urinary incontinence occurred in 31% of patients one week after catheter removal, higher than some reports (24). However, by 12 weeks, incontinence had resolved in all patients, a notably better outcome than in similar studies. This may be attributed to the preservation of the apical mucosa during surgery, which likely contributed to the reduced incidence of long-term incontinence.

During the plateau phase, one case had an extended operation time of 290 min due to the morcellator's inability to effectively fragment the tissue. Upon inspection, the morcellator blade was sharp and showed no signs of wear, suggesting that the difficulty in fragmentation may have been caused by unusually hard prostate tissue (25). There is limited literature on the detection of prostate tissue hardness, indicating the need for further research. We plan to closely monitor similar cases to identify common factors and potential prognostic risks, with the aim of organizing a larger study for further investigation.

Some studies have reported on enucleation time-energy efficacy, defined as enucleated weight divided by enucleation time and consumed energy (17). In our initial analysis, we observed that novice surgeons tended to use lower overall power compared to their more experienced counterparts, likely due to differing usage habits. As a result, enucleation time-energy efficacy was not included as a statistical outcome in this study.

This study included 100 patients, but some did not adhere to follow-up after being satisfied with their surgical outcomes, limiting accurate postoperative Qmax data to 38 cases. Among these, two patients showed a postoperative decline in Qmax. Preoperative values were 8.2 ml/s and 9.5 ml/s, with post-void residuals (PVRs) of 80 ml and 110 ml. One month later, Qmax had dropped to 6.9 ml/s and 5.6 ml/s, with PVRs of 20 ml and 40 ml, and reduced total urine volumes of 72 ml and 55 ml. Despite these decreases, both patients saw significant improvements in IPSS scores. We believe the Qmax reduction is likely due to low bladder volume rather than surgical issues. Future studies will aim to improve follow-up and further investigate the reasons for reduced bladder capacity by examining changes in voiding and storage phases through IPSS analysis.

Combining both domestic and international research along with our own experience, laser enucleation of the prostate is bound to become a simple, easy-to-learn, and universally applicable surgical method that will be widely adopted both domestically and internationally. Of course, whether different energy mediums are suitable for prostate enucleation surgery requires personal verification by every urologist. From our study, it appears that the thulium laser fiber is a qualified energy medium for enucleation and is readily accepted by urologists.

In our study, although the ThuFLEP offers distinct advantages, it is undeniable that certain complications—such as early postoperative urinary incontinence and bladder mucosal injury—have not been markedly reduced, a finding consistent with previous reports and pooled analyses. Consequently, establishing longer-term follow-up protocols and conducting more thorough, accurate preoperative counseling are essential (26).

## 5 Conclusion

Transurethral Thulium Fiber Laser Enucleation of the Prostate (ThuFLEP), with preservation of the urethral mucosa at the prostatic apex, shows clear benefits, including improved operative efficiency and enucleation efficacy. The learning curve reaches proficiency after 50–60 cases, with significant improvements in operative time and efficiency. Postoperative outcomes, such as IPSS improvement and urinary incontinence recovery, further highlight the technique's safety and effectiveness. Early identification and management of potential complications, such as bladder mucosal injury and postoperative bleeding, are crucial. Structured mentorship greatly enhances the learning process, ensuring its broader clinical adoption.

## Data Availability

The original contributions presented in the study are included in the article/Supplementary Material, further inquiries can be directed to the corresponding author.
